# Tip relief designed to optimize contact fatigue life of spur gears using adapted PSO and Firefly algorithms

**DOI:** 10.1007/s42452-020-04129-4

**Published:** 2021-01-11

**Authors:** Raynald Guilbault, Sébastien Lalonde

**Affiliations:** Department of Mechanical Engineering, École de technologie supérieure, 1100 Notre-Dame Street West, Montreal, QC H3C 1K3 Canada

**Keywords:** Gear dynamics, Contact fatigue, Profile modifications, Optimization, Metaheuristic algorithms

## Abstract

This paper examines the dynamic performances of circular profile modifications designed to optimize the contact fatigue life of spur gears. It combines the PSO and Firefly metaheuristics to a gear dynamic/degradation model. The objectives are to analyse the ability of optimal corrections to reduce dynamic loads and dynamic transmission error (DTE), and to describe the influence of the modification variables. To reduce computation efforts, the study modifies the original metaheuristics. In the proposed adaptation of the Firefly algorithm, the particle movement hinges on the brightest firefly perceived through the light-absorbing medium. This change reduces the number of function evaluations per iteration. The analysis shows that while the correction length is more influential, both modification amount and length alter the gear behavior, whereas the curvature radius influence remains modest. Curved corrections are more effective in ameliorating contact fatigue life, whereas larger curvature radii are better at reducing the DTE. Compared to the original gear set, the PSO and Firefly versions showed that optimized modifications engender substantial enhancements of the fatigue resistance. Moreover, optimal profiles also reduce both DTE and dynamic factors, but the inverse cannot be assumed.

## Introduction and literature survey

This paper revisits the concept of gear tooth profile modification optimized to improve the performance of spur gears. The investigation considers the optimization problem from a contact fatigue perspective; an optimal profile should minimize the number of contact subsurface micro-crack initiation points. In other words, it should retard the formation of spalling and pitting craters. To handle the optimization task, the study adopts a metaheuristic optimization approach.

Gear tooth profile modifications have become an industrial routine. While tip and root relief have similar effects, this work mainly concentrates on tip relief. These adjustments are known to reduce Static Transmission Error (STE) as well as Dynamic Transmission Error (DTE) [1] and dynamic loads, and, as a result, gear noise [2]. Lin et al. [2] demonstrated that the dynamic factor Kv (defined as the ratio between the maximum dynamic load to the static load) is correlated with an effective transmission error calculated from the first Fourier harmonic components of the STE. They also showed that for a given design load there is a modification length for any modification amount that minimizes the dynamic load, and that one of those combinations is optimal. The tip relief beneficial effects have also been clearly demonstrated by experimental measurements [3]. In fact, as reported in [2], according to a survey published by D. B. Welbourn in 1979, safe and relatively precise design guidelines are already in place. Built on the combined tooth deflection at the highest point of single tooth contact (HPSTC), these recommendations clearly reveal the ascendance of the operation point; a profile modification optimal for a given load and speed combination is probably not optimal for different operation conditions [4, 5]. Accordingly, the authors of Ref. [5] presented a central investigation on the effects of linear profile modifications and proposed a design procedure to minimize the dynamic factor for high-contact ratio gears submitted to load ranges. A linear modification provides a continuity of order C^0^. Therefore, these authors compared in [3, 6] the effects of linear profile corrections to parabolic modifications applied to low-contact ratio gears. These effects assure a continuity order C^1^ or of both the modification and its first derivative, which means that at its beginning the modification remains tangent to the original profile [3]. While the authors did not notice any measurable advantage of the parabolic shape in Ref. [3], in [6] they observed that, depending on the operating conditions, either the linear or the parabolic options can be effective. They also reported that regardless of the modification type, the correction length has a greater effect on gear dynamic responses than the amplitude of the profile modification.

While Refs. [3, 6] did not consider the fatigue contact aspect of the problem, the authors did assume as a necessary consequence that reducing dynamic loads would also improve pitting fatigue life [6]. This assumption is probably not sure-enough, since fatigue degradation proceeds from high cycle compressive and non-proportional stresses below the contact surfaces [7]. Fatigue degradation therefore depends not only on the dynamic loads, but also on the curvature radii of the contact pair. Tooth profile modification affects both the dynamic loads and the form of the surfaces bearing them. It consequently seems reasonable to include an additional geometric variable into the analysis of profile modifications, instead of restricting the investigation to dynamic loads. While to the best of the authors’ knowledge, no thorough study has been published on the subject, documents like the brief section on involute profile modification in the ANSI/AGMA 6002-C15 Standard [8], which makes a clear distinction between the contact fatigue aspect and the other elements altered by profile modifications, sustain this view. In fact, publication such as [9], which compares the quasi-static contact pressures resulting from circular and linear modifications, have already revealed the surface shape influence: linear modifications introduce discontinuities at the transition points between the modified and the original profile leading to contact pressure rises.

This succinct description brings to light the interactions between the variables of the problem; in essence, the design of a globally performant profile modification corresponds to an optimization problem.

Tavakoli and Houser [10] were among the first to integrate an optimization algorithm into the study of profile modifications. Their investigation makes use of an algorithm combining the Box method to the steepest descent method and a uni-variable search. The authors considered both tip and root relief along with linear and parabolic corrections. In order to reduce gear noise, they defined an objective function corresponding to the sum of the amplitude of the first three harmonics of the STE. The authors emphasized the fact that this multivariable optimization problem definition results in multimodal landscapes. A later work [11] compared an objective function composed of DTE evaluations made over a range of load-speed combinations to a second objective function determined from STE estimates established for the same load range. The optimization algorithm put forward in [11] combined a random search to the Simplex method. The study considers both linear and parabolic profile corrections. This investigation shows that a strategy based on DTE is better at improving gear dynamic responses. Reference [11] also demonstrates the multimodal nature of the problem, and that the number of minima is larger when considering the DTE. More recently, Ref. [12] analysed the influence of pitch and profile errors in parallel with load intensity variations on the definition of optimal linear profile modifications. The proposed optimisation strategy proceeds from graphical searches based on dynamic forces calculated over a speed range. The reference results indicate that, while the studied tooth errors significantly affect the optimal proportions of the corrections, the load variations play a negligible role.

Although the metaheuristic algorithm (MA) avenue seems adapted to the complexity of the problem of gear profile optimization, the literature mainly offers studies proposing combinations of classic deterministic methods. Reference [13] is among the few publications applying MAs to the optimization of gear correction design. The study proposes the optimisation of linear and parabolic corrections based on Genetic Algorithms (GAs). The authors evaluated the sensitivity of the optimal designs resulting from this strategy to manufacturing deviations and concluded that the GA approach is able to provide robust optima.

Reference [14] presents a comprehensive review of most of the recent MA investigations. The author points out that while effective, GAs have a few disadvantages. One of these is that the parameter settings are very delicate [15]. Alternatively, the Particle Swarm Optimization algorithm (PSO) replaces the GA parameters by random real numbers and global interactions among particles, reducing the parameter tuning complexity, which results in a simple, easy to implement and highly efficient algorithm [14]. Essentially PSO deploys a social particle swarm to probe the search space and identify a global optimum. Also based on swarm intelligence, the Firefly algorithm (FA) was developed by Yang in 2008 [16]. The author demonstrated, by means of selected benchmark functions, that the FA could outperform GAs and PSO. In PSO, the particle position improvement is mainly driven by the position of the most optimal particle of the swarm [17]. In the FA, the amelioration of the particle positions are instead guided by better particles located in visible positions in the area surrounding the particle [16]. The swarm can therefore easily split up in clusters and converge onto different local optima encompassing the global optimum [16]. This capacity is especially advantageous in multimodal landscapes.

This portrayal indicates that, while both are potentially efficient, either the linear or the parabolic profile modifications fully outperforms the other, and that their response follows from a balance between their dimensions (correction amplitude and length). It also suggests that the modification designs built on information obtained from dynamic estimates produce corrections that are more effective. Therefore, the present investigation concentrates on dynamic evaluations. This study only considers tip relief defined by circular modifications. The selected definition of the correction includes the linear modification possibility.

The first objective of the paper is to analyse the performance of profile corrections designed to optimize contact fatigue life in terms of their ability to reduce dynamic loads and DTE amplitudes. As a second objective, the study aims at illustrating the influence of the variables defining a circular correction: the starting point, the correction amount and the curvature radius, as well as their interactions.

This work makes use of a contact degradation model coupled with a gear model we developed and recently published [18]. This representation includes a prognosis of contact crack initiation combining multiaxial fatigue criteria with a damage accumulation procedure accounting for the load history. The present study associates this gear model with PSO and FA to determine the optimal amplitude of the variables defining a circular profile correction for given gear sets. Moreover, since the optimization of dynamic and cumulative processes leads to demanding computational efforts, the paper proposes some adaptation of the algorithms to alleviate the expensive calculation.

## Summary of the gear model

**Fig. 1:**
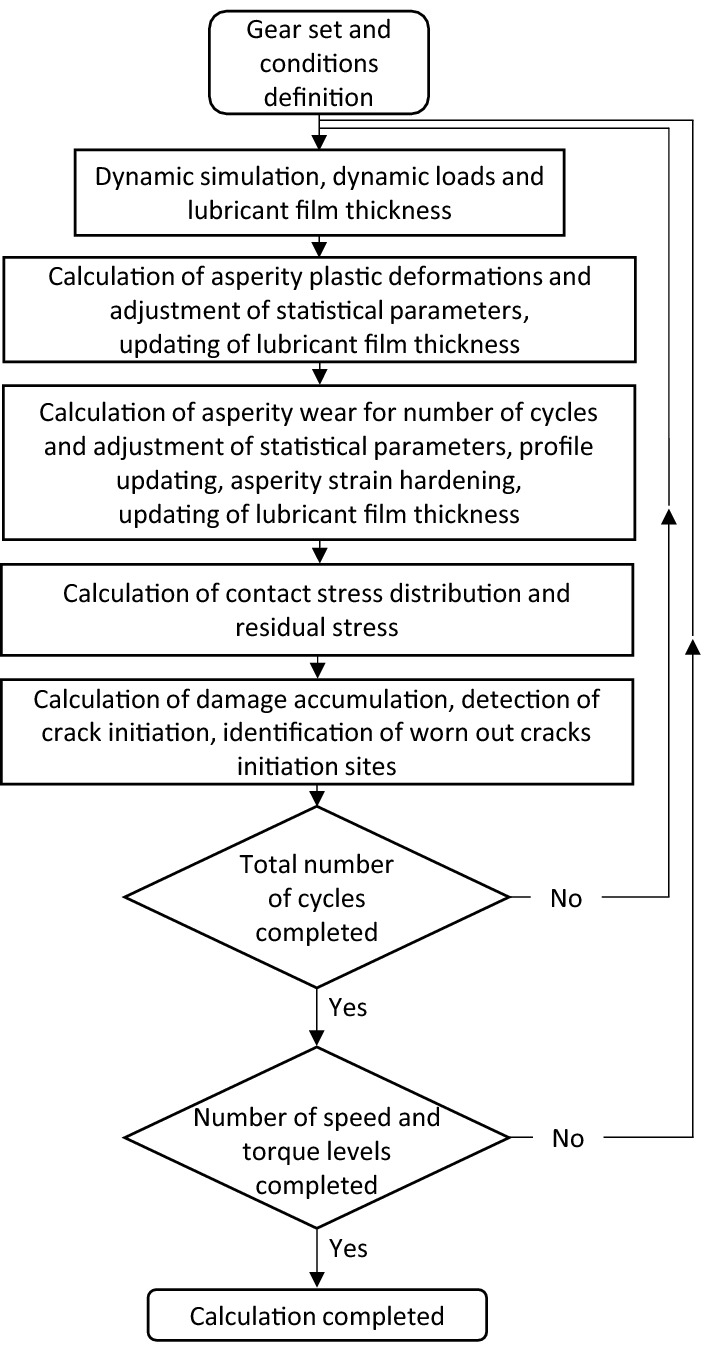
Model flowchart

Figure 1 presents the flowchart of the whole gear dynamic–degradation model. References [19, 20] offer a complete description of the gear non-linear dynamic modeling part. Therefore, since the present paper concentrates on the optimization aspect of the profile correction design, the gear non-linear dynamic modeling segment is not detailed here. Moreover, Ref. [18], which incorporates the contact degradation model into the non-linear dynamic simulations, provides a full description and a complete validation of the contact degradation calculation. Hence, the following only recapitulates the important aspects of the contact degradation model.

First, at each mesh position, the dynamic model provides the individual tooth pair rigidity, the pressure distributions, as well as the total dynamic force. It considers all the profile corrections and possible fabrication errors, along with the evolving modifications associated with damage progression. The calculation of the load sharing between the tooth pairs includes all of those elements. The model divides the tooth profiles into surface segments and realizes the computations for each segment. In addition to the contact pressure, the dynamic part provides the lubricant film thickness between rough surfaces, the contact losses and the slipping distances. The subsequent phases establish the asperity deformation and strain hardening as well as the wear progression. The process then determines the contact stress distributions and the possible residual stress generation. The resulting distributions are then verified for their impact on contact fatigue together with crack growth at the tooth roots. The damage evolution determined for the considered cycles accumulates at the monitored contact crack initiation points below the profile surfaces or at the tooth roots for bending cracks. The present study only includes the contact fatigue aspect, and neglects the root cracks, surface wear and asperity hardening elements.

The rolling contact fatigue problem is associated with out-of-phase multiaxial stresses. Therefore, the model developed in [18] integrates a fatigue contact prognosis via three multiaxial high-cycle fatigue criteria (Dang Van, Liu Mahadevan and Papadopoulos). The analysis presented in [18] demonstrates that the Liu–Mahadevan and the Papadopoulos criteria are better adapted to the gear problem and provide equivalent predictions. Therefore, since there is no need for duplicate appraisals, the present study only considers the Liu–Mahadevan criterion developed in [21].

The Liu–Mahadevan criterion is based on the critical plane approach. It assumes that cracks should initiate on a critical plane equivalent to a crystal slip plane, and thereafter propagate along a fracture plane perpendicular to the maximum principal stress direction. The criterion is adapted to the present problem by Eq. (1), where *Ω* is the angle formed by the critical plane with the fracture plane. This angle depends entirely on the material properties. The variables *S*_*a*,*c*_ and *τ*_*a*,*c*_ are the normal and shear stress amplitudes active on the critical plane, *S*_*m,c*_ is the mean stress component on the critical plane, and *S*_*lim,Nf*_ and *τ*_*lim,Nf*_ are the fatigue limits for *N*_*f*_ cycles to failure in fully reversed bending and torsion, respectively. These fatigue limits and related cycles to failure are established from Stress-cycle (*SN*) or Wöhler curves.

1-a $$\frac{1}{B}\sqrt {\left( {\frac{{\tau_{{lim,N_{f} }} }}{{S_{{lim,N_{f} }} }}} \right)^{2} \left[ {S_{a,c} \left( {1 + \eta_{{N_{f} }} \frac{{S_{m,c} }}{{S_{{lim,N_{f} }} }}} \right)} \right]^{2} + \left( {\tau_{a - c} } \right)^{2} } \le \tau_{{lim,N_{f} }}$$

1-b $$\Omega = \frac{1}{2}\cos^{ - 1} \left( {\frac{{ - 1 + \sqrt {1 - \left( {{1 / {s_{r}^{2} - 3}}} \right)\left( {{{5 - 1} / {s_{r}^{2} - 4}}s_{r}^{2} } \right)} }}{{\left( {{{5 - 1} / {s_{r}^{2} - 4}}s_{r}^{2} } \right)}}} \right)$$

1-c $$B = \sqrt {s_{r}^{2} \cos^{2} (2\Omega ) + \sin^{2} (2\Omega )}$$

1-d $$\eta_{{N_{f} }} = \frac{3}{4} + \frac{1}{4}\left( {\frac{{\sqrt 3 - \frac{{S_{{\lim ,N_{f} }} }}{{\tau_{{\lim ,N_{f} }} }}}}{\sqrt 3 - 1}} \right)$$

1-e $$s_{r} = \frac{{\tau_{{\lim ,N_{f} }} }}{{S_{{\lim ,N_{f} }} }}$$

### Damage accumulation

The model integrates a damage accumulation indicator presented by Mesmacque et al. [22]. This damage rule is simple and works with Wöhler curves. Moreover, the analysis presented in [18] indicates that, compared to other damage accumulation approaches, this indicator demonstrates better overall suitability. Equations (2)–(4) formulate this damage accumulation rule.

The damage parameter *D*_*j*_ describes the actual damage level at time position *j*: *D*_*j*_ = 0 for an intact material and *D*_*j*_ = 1 at failure. Equation (2) defines *D*_*j*_, where *S*_*ut*_, *S*_*a,j*_ are the tensile strength and the stress amplitude repeated *N*_*cc*_ times during the time cluster *j*, respectively. Also, if *S*_*a,j*_ corresponds to *N*_*f,j*_ cycles to failure and *N*_*f,j*_ ≥ *N*_*cc*_, *S*_*lim,*(*Nf,j*–*Ncc*)_ represents the fatigue limit for the remaining life (*N*_*f,j*_ – *N*_*cc*_) cycles:

2 $$D_{j} = \frac{{S_{{lim,(N_{f,j} - N_{cc} )}} - S_{a,j} }}{{S_{ut} - S_{a,j} }}$$

After *N*_*cc*_ cycles at *S*_*a,j*_ or at the beginning of the next cycle cluster *j* + 1, *D*_*j*_ translates as indicated by Eq. (3), where *S*_*eq*_ describes an equivalent fatigue limit associated with *N*_*f,j*+1_ cycles:

3 $$D_{j} = \frac{{S_{eq} - S_{a,j + 1} }}{{S_{ut} - S_{a,j + 1} }}$$

Therefore, after *N*_*cc*_ cycles of cluster *j* + 1, *D*_*j*+1_ becomes [Eq. (4)]:

4 $$D_{j + 1} = \frac{{S_{{\lim ,(N_{f,j + 1} - N_{cc} )}} - S_{a,j + 1} }}{{S_{ut} - S_{a,j + 1} }}$$

Equations (3) and (4) describe the damage progression up to failure as long as *D*_*j*+1_ < 1. In the present analysis failure means the initiation of a contact crack. The relation between *S* and *N*_*f*_ proceeds from the material Wöhler curve. This represents a real advantage, since no additional material properties are required.

The contact fatigue model combines the Liu–Mahadevan multiaxial high cycle fatigue criterion given by Eq. (1) with the damage calculation formulated by Eqs. (3) and (4).

**Fig. 2:**
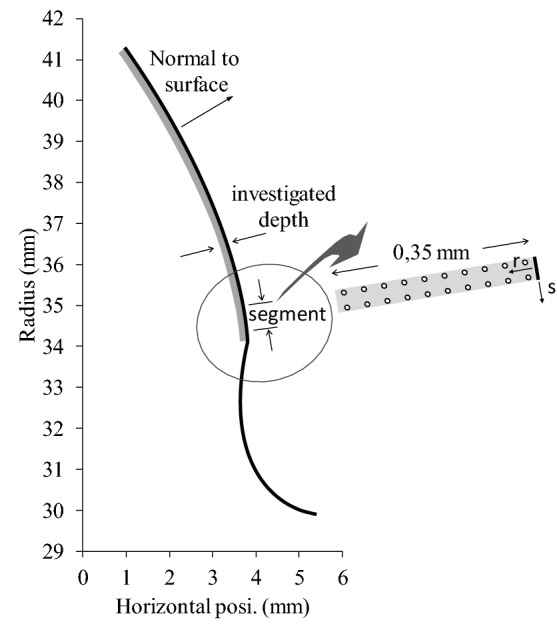
Pinion profile and surface layer monitored for contact crack initiation detection

The process follows selected positions in a subsurface material layer for each tooth of the gear pair. Figure 2 shows the complete profile shape of the pinion teeth of the gear pair investigated later in this study. In addition to the tooth shape, this figure also illustrates the monitored subsurface layer. For each segment of the tooth profiles, two rows of 10 points are monitored for contact damage accumulation. The points are aligned with the normal to the middle of each segment. In the present investigation the profiles are divided into 500 segments; the simulations monitor 10 × 10^–3^ potential contact crack initiation points for each tooth.

### Contact and residual stress

#### Contact stress

**Fig. 3:**
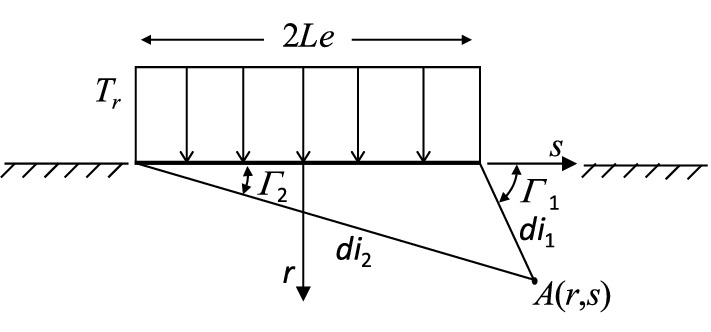
Tooth profile segment of constant traction (*T*_*s*_ = 0)

The contact model divides the contact pressure distribution into a collection of *l* profile segments of length 2*Le* supporting constant traction *T*_*r*_ and *T*_*s*_ in the normal and tangential directions, respectively. Therefore, from [23], Eqs. (5) and (6) give the stress values at any position *A*(*r*, *s*) in a tooth body (see also Fig. 3):

5a $$S_{rr} = - \frac{{T_{r} }}{2\pi }\left[ {2\left( {\Gamma_{1} - \Gamma_{2} } \right) - \left( {\sin 2\Gamma_{1} - \sin 2\Gamma_{2} } \right)} \right]$$

5b $$S_{ss} = - \frac{{T_{r} }}{2\pi }\left[ {2\left( {\Gamma_{1} - \Gamma_{2} } \right) + \left( {\sin 2\Gamma_{1} - \sin 2\Gamma_{2} } \right)} \right]$$

5c $$S_{rs} = - \frac{{T_{r} }}{2\pi }\left[ {\cos 2\Gamma_{1} - \cos 2\Gamma_{2} } \right]$$

6a $$S_{rr} = - \frac{{T_{s} }}{2\pi }\left[ {\cos 2\Gamma_{1} - \cos 2\Gamma_{2} } \right]$$

6b $$S_{ss} = - \frac{{T_{s} }}{2\pi }\left[ {4\ln \left( {\frac{{di_{1} }}{{di_{2} }}} \right) - \left( {\cos 2\Gamma_{1} - \cos 2\Gamma_{2} } \right)} \right]$$

6c $$S_{rs} = - \frac{{T_{s} }}{2\pi }\left[ {2\left( {\Gamma_{1} - \Gamma_{2} } \right) + \left( {\sin 2\Gamma_{1} - \sin 2\Gamma_{2} } \right)} \right]$$

where $$\tan \Gamma_{1} = \frac{r}{s - Le}$$, $$\tan \Gamma_{2} = \frac{r}{s + Le}$$ and $$di_{1} = \sqrt {r^{2} + \left( {s - Le} \right)^{2} }$$, $$di_{2} = \sqrt {r^{2} + \left( {s + Le} \right)^{2} }$$.

Assuming a plain strain state condition, Eq. (7) gives the principal shear stress (*τ*_*max*_).

7 $$\tau_{\max } = \sqrt {\frac{1}{4}\left( {S_{ss} - S_{rr} } \right)^{2} + S_{rs}^{2} }$$

The stress amplitudes required for the multiaxial fatigue criterion should normally call for a loading cycle counting method. However, the nature of the dynamic tooth contact essentially leads to one loading cycle over a mesh period, and this cycle is defined by a minimum value fixed by the maximum contact load intensity and a maximum value of zero. Therefore, the model does not incorporate a cyclic counting method.

#### Residual contact stress

In order to maintain acceptable calculation times, the developments presented in [18] assume that the plastically deformed zones, as well as the residual stresses, may be established from the elastic Hertz theory. Therefore, with the assumption of plane strain deformation, and considering a perfectly plastic material, residual stress distributions (*ρ*_*ss*_) develop only in the *s* direction to compensate for yield according to the Tresca criterion.

After calculation of the elastic stress state with Eqs. (5) and (6) based on the Tresca criterion, Eq. (8) formulates *ρ*_*ss*_. This residual stress evaluation is cumulative.

8 $$\rho_{ss} = \left\{ {\begin{array}{*{20}l} {2\sqrt {k_{y}^{2} - S_{rs}^{2} } + \left( {S_{rr} - S_{ss} } \right)} \hfill & {{\text{if}}\;\;\left| {S_{rs} } \right| \le k_{y}^{{}} } \hfill \\ {\left( {S_{rr} - S_{ss} } \right)} \hfill & {{\text{if}}\;\;\left| {S_{rs} } \right| > k_{y}^{{}} } \hfill \\ \end{array} } \right.$$

For each segment, the stress (elastic and residual) calculation procedure goes over 20 monitored points uniformly arranged in two columns in the *r* direction (underneath the surface, Fig. 2). While the investigated depth is left as an adjustable parameter, the present analysis considers a layer depth of 0.35 mm.

## Metaheuristic algorithms—PSO and Firefly

The literature shows that the optimization space defined by the tip relief of gear teeth is potentially multimodal, and that the multimodality of the problem tends to increase when considering dynamic parameters. While the present study does not explore the multimodal nature of the problem, it seems pertinent to adopt an approach designed to navigate over such landscapes. In addition, since for the investigated design problem, it remains conceivable that multiple minima provide efficient response, Firefly, which can simultaneously converge toward multiple minima, offers the required capacities. However, finding the global optimal definition of a profile correction remains the aim, and thus PSO, which focuses on finding the global optimum, still remains an appropriate option.

Because of the accumulative nature of contact fatigue requiring iterative calculations at numerous surveyed points, leading to time-demanding simulations, the adopted optimization process does not include the usual repetition of the searches. However, both PSO and Firefly algorithms independently identify an optimal tip relief design, and the comparison of the obtained solutions allows substantiating the results.

### PSO

The standard PSO algorithm rests on a searching swarm composed of *n* particles located at various positions over the landscape. Each of these particles may potentially be situated close to an optimal location. At iteration *t* + 1 the particle *i* search progresses towards an optimum of the *D* dimensional space according to its own best position *p*_*best i*_ encountered so far, which corresponds to a memory term, and the swarm global best position *g*_*best *(*t*)_ found during iteration *t*. This second term introduces the social knowledge. The influence of these contributors is modulated by random parameters to bring in a certain balance between the space exploration and the knowledge exploitation. The calculation of the position change also includes the particle inertia. Equation (9) formulates this description and gives the velocity *v*_*id *(*t*+1)_ of particle *i* for dimension *d* out of *D* at iteration *t* + 1. In Eq. (9) *W* is the constant inertial weight of the previous particle advance (0 ≤ *W* ≤ 1), *c*_1_ and *c*_2_ are acceleration constants (≈ 2), and *ε*_1 (*t*)_ and *ε*_2 (*t*)_ are random numbers between 0 and 1. Equation (10) gives the new position *x*_*id* (*t*+1)_ of particle *i* for dimension *d*.

9 $$v_{id\,(t + 1)}^{{}} = Wv_{id\,(t)}^{{}} + c_{1} \varepsilon_{1\,(t)}^{{}} \left( {g_{best\,d\,(t)}^{{}} - x_{id\,(t)}^{{}} } \right) + c_{2} \varepsilon_{2\,(t)}^{{}} \left( {p_{best\,id}^{{}} - x_{id\,(t)}^{{}} } \right)$$

10 $$x_{id\,(t + 1)}^{{}} = x_{id\,(t)}^{{}} + v_{id\,(t + 1)}^{{}}$$

**Fig. 4:**
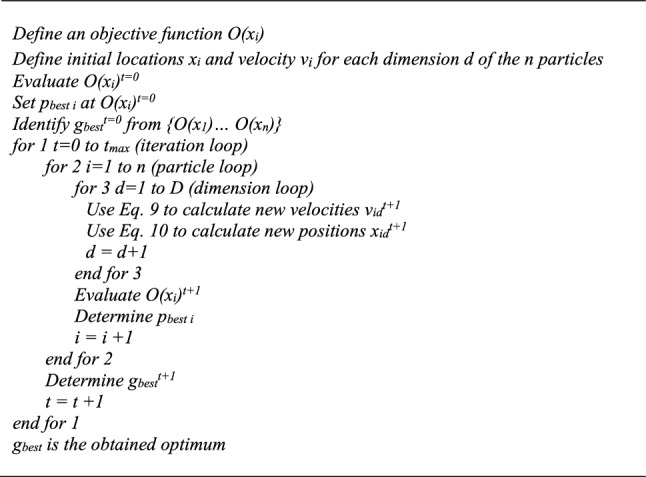
Standard PSO pseudo code

Figure 4 presents the pseudo code of the standard PSO algorithm.

In addition to its simplicity, PSO also provides fast search speeds, since only the information from a unique best particle can be transferred to the next generation [24]. On the other hand, this strategy may cause the algorithm to stop at local optima. Therefore, to reduce the premature convergence probability, the random numbers maintain a given level of exploration, which also has the consequence of causing some jumping around the space optimum. To reduce this side effect, the contribution of the random numbers in Eq. (9) may be decreased with the iteration progress [14]. The inertia introduced in Eq. (9) also causes *g*_*best*_ to overfly the space optimum.

To reduce the overflying effect of *g*_*best*_ while preserving the exploration capacity of PSO, this study modifies the algorithm of Fig. 4 to transfer the *g*_*best*_ of iteration *t* to iteration *t* + 1. The following additional line is introduced in the pseudo code in-between the particle loop *for* *2* and the dimension loop *for 3*: “*if x*_*id *(*t*)_ = *g*_*best *(*t*)_, *then W* = *0, otherwise W* = *k”*, where *k* is set based on the literature at 0.729.

### Firefly

**Fig. 5:**
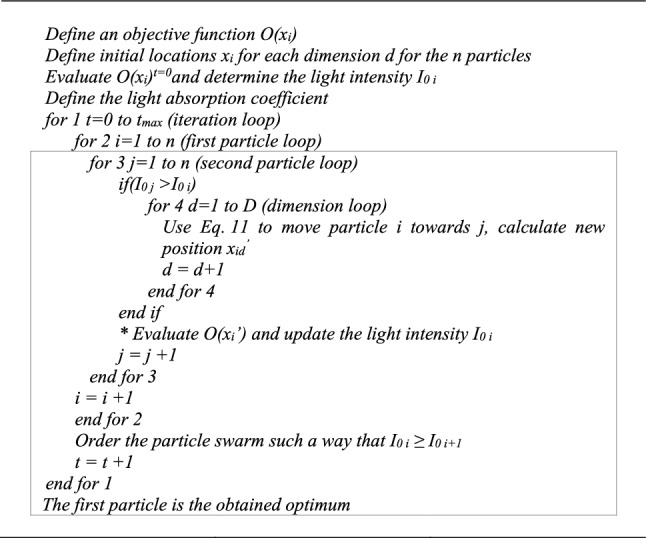
Original Fa pseudo code

As PSO, the Firefly algorithm (Fa) also counts on a searching swarm composed of *n* particles. Fa was first introduced by Yang 16]. The algorithm assumes that all fireflies are genderless and may be attracted by any other firefly. The attractiveness *β* of a firefly is commensurate with the intensity of its light emission (*I*) seen by another firefly located at a distance *r*, where *r* is the Euclidian distance between particle *i* and a second particle *j* $r_{ij} = \sqrt {\sum\nolimits_{d = 1}^{D} {\left( {x_{jd} - x_{id} } \right)^{2} } }$. If *I*_0_ is the particle light intensity at the source, at a distance *r* in a medium with a light absorption coefficient *γ*, then *I* correspond to $I(r) = I_{0} e^{{ - \gamma r^{2} }}$. The light intensity *I*_0_ of particle *i* results from its response to the objective function *O*(*x*_*i*_). Thus, the attractiveness becomes $\beta (r) = \beta_{0} e^{{ - \gamma r^{2} }}$, where *β*_0_ corresponds to the particle attractiveness at the source. Therefore, the position change of particle *i* attracted by a brighter particle *j* is given by Eq. (11):

11 $$(x_{id}^{{}} )^{\prime} = x_{id\,(t)}^{{}} + \beta (r)\left( {x_{jd\,(t)}^{{}} - x_{id\,(t)}^{{}} } \right) + \alpha S_{d} \varepsilon$$

where *αS*_*d*_*ε* is a random contribution forcing the space exploration, *α* is the random parameter, and *S*_*d*_ is a scaling factor adapting the random influence to the scale of the considered dimension *d*. *S*_*d*_ also assures the dimension homogeneity. It corresponds to the difference between the upper and lower limits of each dimension *d*. *ε* is a random number between − 0.5 and 0.5. In [16] Yang suggested that $\beta_{0} \simeq 1$, $\alpha \in \left[ {0,1} \right]$, and $\gamma \in \left[ {0.001,1000} \right]$. Low values of *γ* are the equivalent of clear skies with high visibility of the particles. On the other hand, high *γ* values diminish the attractiveness of the fireflies, and thus reduce the search to a random roaming of the particles. Figure 5 presents the pseudo code of the original Fa.

The pseudo code in Fig. 5 shows that if we assume that after ordering the swarm, the light intensity of particle *i* cannot decrease below the light intensity of particle *i* + 1, the strategy could require up to $0.5n(n - 1)$ evaluations of *O*((*x*_*i*_)′) at each iteration. In reality, during the process, this assumption on the light intensity is far from being sure, and the final evaluation number could be larger. Therefore, while clearly reliable, depending on the function estimation time, this approach may result in significant computation efforts. However, even more pivotal is the indefinite nature of this course of action, which corresponds to unpredictable durations. Therefore, to improve the manageability of the process, the evaluation line identified by a * in Fig. 5 may simply be moved below the *end for 3* line. Studies such as [25, 26] demonstrated the capability of this algorithm, which while slightly diminishing the precision of the process, does reduce the function evaluation number per iteration to *n*.

**Fig. 6:**
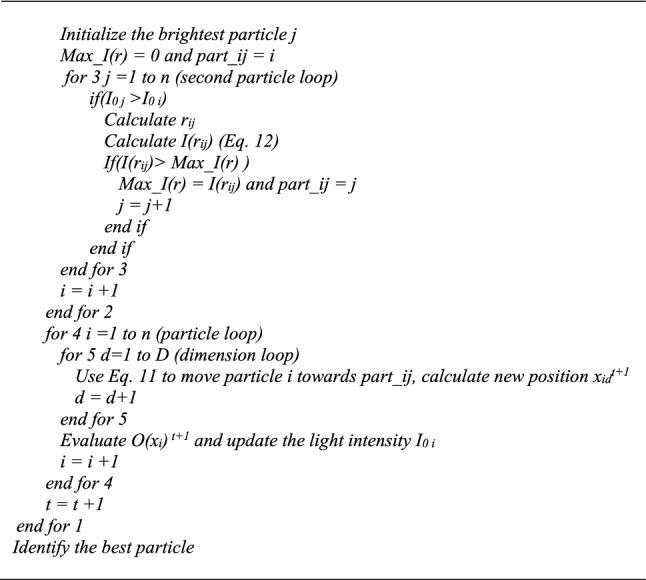
Pseudo code of the modified section of the original Fa

Since the optimisation problem in the present study involves contact fatigue degradation occurring in dynamic systems, the function evaluations are computationally demanding. Thus, in order to preserve the advantages of both algorithm variants, we instead opted for a trade-off strategy, where the movement of a particle *i* results from the attraction of only one particle *j* at each iteration. This attractive particle *j* is the brightest firefly perceived by particle *i* at a distance *r*_*ij*_. Therefore, among all particles *j* satisfying the condition (*I*_0* j*_ > *I*_0* i*_), the brightest one for firefly *i* is the one maximizing $I(r_{ij} ) = I_{0} e^{{ - \gamma r_{ij}^{2} }}$. Following this approach, the function evaluation number per iteration remains *n*, and so the ordering of the firefly swarm is no longer required. Moreover, to maintain the dimension homogeneity, *γ* should have units inverse to those of *r*_*ij*_. Hence, in the proposed Fa adaptation, *γ* is normalised by a representative length (*le*_*rep*_). The best option for *le*_*rep*_ appears to be the landscape diagonal. Equation (12) gives the expression for *I*(*r*_*ij*_). Thus, substituting the lines given in Fig. 6 for the section surrounded by a rectangle in Fig. 5 gives the pseudo code of the modification proposed for the original Fa.

12 $$I(r_{ij} ) = I_{0} e^{{\frac{{ - \gamma r_{ij}^{2} }}{{le_{rep}^{2} }}}}$$

The literature contains numerous variants of the original Fa (see [27] for examples). Both *α* and *β* may be adapted. Decreasing the random parameter *α* while progressing toward the final iterations will favor landscape exploitation. In fact, *α* should be zero when the process converges to an optimum [28]. A common function considered in the literature to introduce the *α* fading is given by Eq. (13), where *α*_0_ is an initial amplitude and $\vartheta$ is a reduction factor, which is often arbitrarily selected at around 0.95 to 0.99 [14].

13 $$\alpha = \alpha_{0} \vartheta^{t}$$

High values of $\vartheta$ generate slow decreases, and thus, dragging transfers from exploration to exploitation of the landscape. On the other hand, lower values such as 0.6 lead to rapid reductions, even putting the search at risk of premature convergence. To maintain a better balance between the landscape exploration and exploitation, the present study introduces a control expression for $\vartheta$, Eq. (14). In Eq. (14) *EPe* represents the chosen ratio $\alpha_{t\max } /\alpha_{0}$ at the last iteration (*t*_*max*_).

14 $$\vartheta = \exp \left( {\frac{{\ln \left( {EPe} \right)}}{{t_{\max } }}} \right)$$

While in the original Fa the attractiveness *β*_0_ is constant and set at 1, many authors (see for example [25–29]) have suggested that variable *β* increases both the success rate and the response precision. Reference [29] evaluated various chaotic maps to generate *β* values. The authors also considered variable absorption coefficient *γ* values generated from the same maps. They showed that tuning *β* is effective, whereas tuning *γ* engenders no significant performance gain. They also observed that the chaotic Gauss/Mouse map given by Eq. (15) offers the best enhancements. Therefore, the present study replaces *β*_0_ in Eq. (11) by a Gauss/Mouse map. Consequently, the following line should be inserted just above the *for 1* line in Fig. 5: “*Use Eq. *(15) *to generate β*_*0*_”:

15 $$\beta_{t + 1} = \left\{ {\begin{array}{*{20}l} 0 \hfill & {{\text{when}}\;\beta_{t} = 0} \hfill \\ {\frac{1}{{\beta_{t} }} - 1\bmod \left( {\beta_{t} } \right)} \hfill & {{\text{otherwise}}} \hfill \\ \end{array} } \right.$$

The authors of [29] tested only chaotic functions, and among them the Gauss/Mouse map, which produces sequences with nearly uniform distribution, generated the best performance improvements. It may thus be conjectured that this property probably explains the reported observations. It is hence reasonable to postulate that a pseudo-random number generator with uniform distribution would be even more effective. However, this aspect is not investigated within the scope of the present analysis; instead, the *β*_0_ values are all generated with Eq. (15).

## Circular tooth profile modification

**Fig. 7:**
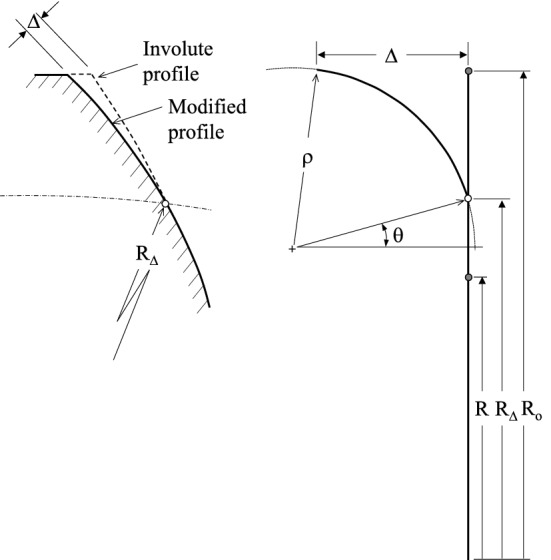
Circular profile modification

Figure 7 illustrates the variable defining circular profile modifications. These variables are *Δ*, the correction amount, the radius *R*_*Δ*_ at the modification starting point, and *ρ*, the curvature radius. As Fig. 7 indicates, this definition does not impose any tangency condition at the correction/original profile junction. Therefore, *Δ*, *R*_*Δ*_ and *ρ* are the sole dimensions considered during the optimisation process, while the angle *θ* is a dependant variable given by Eq. (16), where $l = \sqrt {\Delta^{2} + \left( {R_{o} - R_{\Delta } } \right)^{2} }$ and *R* and *R*_*o*_ are the gear pitch and outside radii. This definition shows that the condition *ρ* → ∞ corresponds to the common linear modification. It also implies that *ρ* must have a minimum value. Equation (17) gives this minimum, and will thus constrain the optimization problem.

16 $$\theta = \cos^{ - 1} \left\{ {\frac{{R_{o} - R_{\Delta } }}{l}} \right\} - \sin^{ - 1} \left\{ {\frac{l}{2\rho }} \right\}$$

17 $$\rho \ge \frac{{R_{o} - R_{\Delta } }}{1 - \sin \theta }$$

## Gear definition and simulation

This investigation considers the non-standard gear set examined in [18]. Table 1 below gives the gear set parameters.

**Table 1:** Gear set parameters

Parameters	Pinion	Gear
Tooth numbers	16	24
Module (mm)	4.5	
Pressure angle (°)	20	
Face width (mm)	14	
Addendum modification	0.171	
Center distance (mm)	91.5	
Initial average tooth flank roughness, *R*_*a*_ (μm)	1.44	1.27

It is considered that the gear wheels were manufactured using a rack-shaped cutter. Therefore, the pinion shows some cutting interference starting at a radius value of 34.4 mm. Figure 2 shows the complete profile shape of the pinion teeth.

**Fig. 8:**
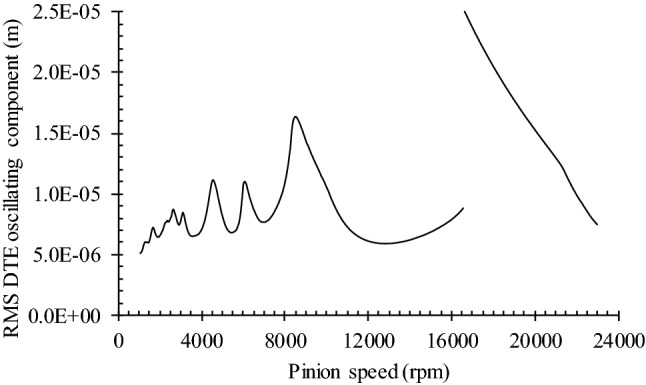
RMS of DTE oscillating component of tested gear pair

Figure 8 describes the gear set dynamic response in terms of its rms Dynamic Transmission Error (DTE) oscillating component for pinions speeds ranging from 1000 to 23,000 rpm when the resistive torque is 148.4 Nm. The graph indicates that the gear pair natural frequency (*ω*_*res*_) occurs around a pinion speed of 16,600 rpm.

The gears are made of carburised steel DIN 20MnCr5 [18]. Table 2 gives the mechanical properties of the material.

**Table 2:** DIN 20MnCr5 mechanical properties (from [18])

Parameters	Pinion and Gear
Elastic modulus, *E* (GPa)	210
Poisson ration, ν	0.3
Yield strength (MPa)	850
Tensile strength (MPa)	1300
Rockwell hardness (HRC)	60

The subsurface fatigue crack initiation simulation requires the material fatigue properties indicated in Table 3 (taken from [18]). These parameters describe a Stress-Cycle (*SN*) or Wöhler curve defined by the common curve fit $S_{f} = a_{W} \left( {N_{f} } \right)^{{b_{W} }}$, here *a*_*W*_ and *b*_*W*_ are constants specific to each material.

**Table 3:** DIN 20MnCr5 fatigue parameters

$S_{f\;}$	$a_{W}$ (MPa)	$b_{W}$
Bending		
$S_{{\lim ,N_{f} \;}}$	2409.560	− 0.10134
Torsion		
$S_{{\lim ,N_{f} \;}}$	1445.736	− 0.10134

The study in [18] included a lubricant respecting the DIN 51517 part 3 requirements. The following parameters were introduced into the model: kinematic viscosity at 40° 116.6 cSt and at 100° 16.9 cSt, density at 15° 962 kg/m^3^, and an oil bath temperature of 80°.

The graphic in Fig. 8 shows that the optimal subcritical operation speed range is located in between 65 and 95% of the resonance speed, or $\in \left[ {10{,}790\;{\text{rpm}},\;14{,}770\;{\text{rpm}}} \right]$. Hence, to avoid the influence of erratic dynamic behaviors, the operation speed is set at 13,000 rpm for all optimisation simulations. The objective is to minimise the total number of subsurface initiation points of contact fatigue. The damage accumulation procedure presented in Sect. 2 establishes the damage level on both pinion and gear. The investigated layer depth is 0.35 mm (see Fig. 2). The addition of the individual numbers of initiated cracks (*IFC*) represents the gear pair response. The simulations include 10^6^ pinion cycles apportioned in cluster *N*_*cc*_ of 10^5^ cycles.

The PSO simulations aim at minimizing the objective function. Thus, the objective function *O*(*x*_*i*_) simply corresponds to the total number of subsurface crack initiation points calculated for the gear set and designated as *IFC*. The Fa progression, instead, depends on light intensities at the source *I*_0_. The better particles are those that maximise *I*_0_. *I*_0_ is thus given by Eq. (18).

18 $$I_{o} = \frac{1}{1 + IFC}$$

As indicated above, the adopted optimization procedure does not involve any repetition. Therefore, to ensure that all zones are visited during the search, the initial particle generations are uniformly distributed over the landscape for both PSO and Fa. Considering that three particles along each dimension are required, the total number of particles *n* is 27. The maximum number of iterations, *t*_*max*_, is fixed at 15. Moreover, the corresponding dimensions of the pinion and the gear vary in their concordance. The range for each dimension is defined as: $\Delta \in \left[ {0,50\;\upmu {\text{m}}} \right],\;\rho \in \left[ {{\text{Eq.}}\,{17},20m} \right],R_{\Delta } \in \left[ {R,R_{o} } \right]$. For Fa, Eq. (13) establishes *α*, with *α*_0_ fixed at 0.25 and *EPe* at 0.1. The initial value *β*_*t*=0_ is set at ${\pi / 4}$. Equation (15) provides *β*_0*t*_ during the subsequent iterations. For PSO, *c*_1_ and *c*_2_ are set based on the literature at 1.49455, while *ε*_1 (*t*)_ and *ε*_2 (*t*)_ values are established between 0 and 1 via a pseudo-random generator.

## Results

### Factorial design analysis

The first particle distribution generates an initial swarm. Because it is uniformly distributed, it also describes a three factor-three level (3^3^) factorial design. This structure itself gives access to meaningful information. Hence, it appears pertinent to devote some time dissecting it.

**Fig. 9:**
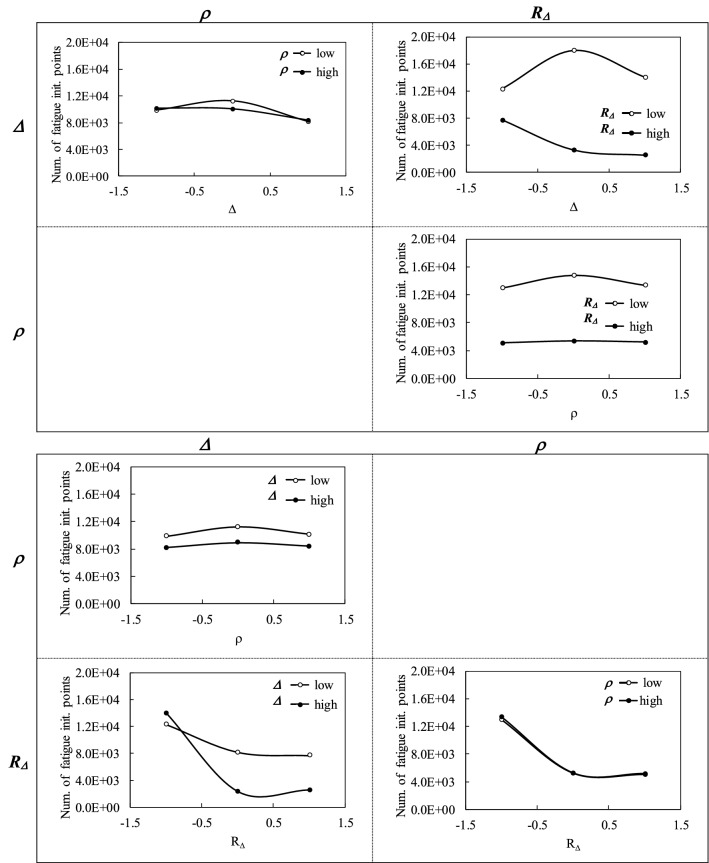
Interaction plots derived from the initial uniform distribution:—the numbers of contact crack initiation points

**Fig. 10:**
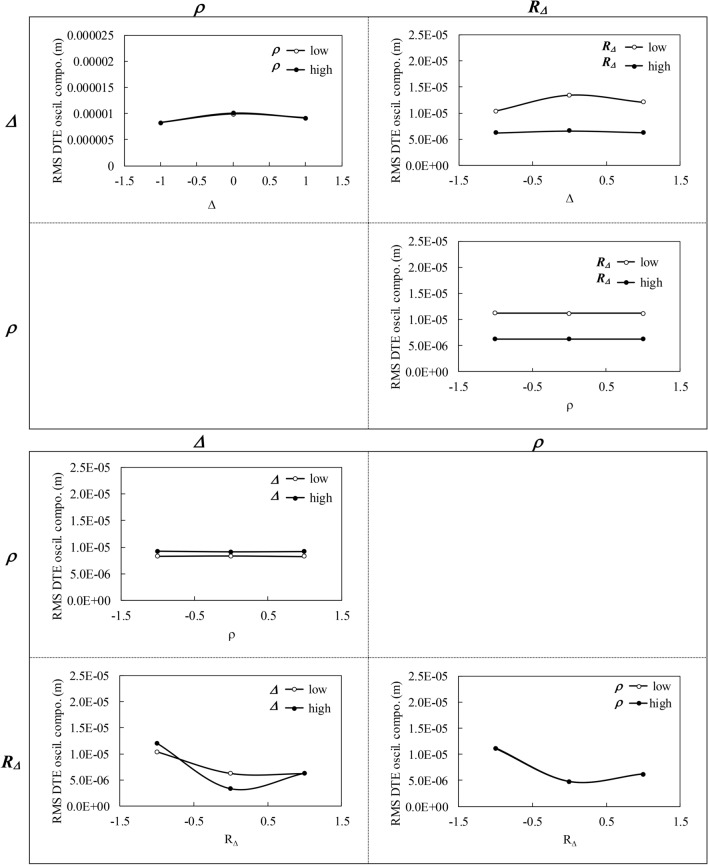
Interaction plots derived from the initial uniform distribution:—the rms DTE oscillating components

The positions along the three factor axes (or the *Δ*, *ρ* and *R*_*Δ*_ dimensions) correspond to one sixth, one half and five sixth of the ranges. Figures 9 and 10 present the two-factor interaction plots prepared from the initial uniform distribution. In addition to the number of points of contact crack initiation shown in Fig. 9, the graphs also display the influence of the factors on the rms DTE oscillating component in Fig. 10. The horizontal axes are normalized; positions − 1 and 1 correspond to the low and high values of the factors, respectively.

The graphs in Fig. 9 reveal that *Δ* and *R*_*Δ*_ may significantly affect the fatigue life of gear sets, and that these factors demonstrate obvious interactions. Greater values of *R*_*Δ*_ or shorter correction lengths better improve the contact fatigue life, particularly when combined with a larger amount of correction *Δ*. Conversely, a too-large *Δ* may increase contact fatigue when associated with high lengths of correction or low *R*_*Δ*_. On the other hand, *ρ* generates less manifest effects, and does not really interact with the two other factors. Overall, smaller *ρ* values leading to curved modifications appear to be slightly more advantageous. Figure 10, which juxtaposes the associated graphs of the DTE oscillating component, shows that the factors engender similar tendencies. However, the amplitude of the changes they induce on the DTE remains low. In particular, the *ρ* plots indicate that modifications of this factor cause no distinct variation. In fact, although shorter correction lengths (high *R*_*Δ*_ amplitudes) are always preferable to improve contact fatigue life, while larger amounts of correction *Δ* are only appropriate with high *R*_*Δ*_, the DTE responses indicate that moderate *R*_*Δ*_ amplitudes are more effective. Also, when combined with moderate correction lengths (moderate *R*_*Δ*_), higher *Δ* values better reduce the DTE component.

Tables 4 and 5 present the calculated values of the factor main effects and of their interactions, respectively. The values in Table 4 corroborate the previous observations: increasing *R*_*Δ*_ and *Δ* reduces the number of fatigue initiation points, whereas modifying *ρ* has little consequences. In particular, Table 4 attests that, lower values of *ρ* are more advantageous. Table 5 brings more precision to *ρ* and reveals that *ρ* final impact marginally depends on *R*_*Δ*_ and *Δ*, although *R*_*Δ*_ demonstrate the highest influence. Table 4 also confirms that the main effects of *R*_*Δ*_ is largely more significant than that of *Δ*. This result agrees with the literature [6]. These findings thus indicate that, while curved profile corrections appear to be slightly more effective in improving the contact fatigue life, the length and the amount of the correction are the controlling parameters. Table 5 also evidences the interactions of these two factors. On the other hand, when considering the DTE response, Table 4 shows that increasing *R*_*Δ*_ still improves the gear set response, whereas the upshots of *Δ* and *ρ* are turned around; increasing the correction amount amplifies the DTE oscillating component, while large *ρ* values tend to better the gear set dynamic response. However, in that case again, the impact of *ρ* remains modest compared to that of *R*_*Δ*_, which is dominant. We thus have to conclude that improving the dynamic response and increasing the contact fatigue life of gear sets are two purposes with requirements that are partially in opposition.

**Table 4:** Factor main effects of the factor on the examined gear set

	*Δ*	*ρ*	*R*_*Δ*_
Number fatigue initiation points	− 1716.3	262.3	− 8024.8
RMS DTE oscillating component (m)	8.83 × 10^–7^	− 3.25 × 10^–8^	− 4.95 × 10^–6^

**Table 5:** Factor interaction effects of the factor on the examined gear set

	*Δρ*	*ΔR*_*Δ*_	*ρR*_*Δ*_	*ΔρR*_*Δ*_
Number fatigue initiation points	− 35.3	− 3431.3	− 132.8	− 132.3
RMS DTE oscillating component (m)	− 1.8 × 10^–8^	− 8.23 × 10^–7^	3.25 × 10^–8^	1.70 × 10^–8^

Based on this factorial design, it becomes simple to anticipate that, centered on contact fatigue, the subsequent optimization process should result in short correction lengths, or rather high *R*_*Δ*_, combined with relatively important correction amounts of *Δ* incorporated in circular modifications.

### Optimization analysis

The optimization procedure involves large cycle clusters *N*_*cc*_ of 10^5^ cycles that affect the precision of the predicted numbers of fatigue initiation points. However, it is worth noting that the optimization strategies do not evolve based on absolute values, but instead on comparisons of particle performances. Consequently, the coarse cycle steps should not really affect the identification of optimum designs. Nevertheless, after defining the optimal profile modifications, in order to provide better estimates of the performances and to compare the original design with the optimized versions defined by PSO and FA, the three gear set responses are re-evaluated considering *N*_*cc*_ of 2 × 10^4^ cycles. All operation conditions remain unchanged.

Table 6 reports the optimization results. The performance re-evaluation step of the original, the PSO optimum and the Fa optimum designs led to *IFC* values of 16.415 × 10^3^, 5.709 × 10^3^ and 6.368 × 10^3^ initiation points of contact fatigue, respectively. These results correspond to improvements of 65.2 and 61.2% for PSO and Fa, respectively. Considering the pinion and gear outside radius of 41.27 and 59.27 mm, the indicated *R*_*Δ*_ defines correction lengths of 2.82 and 2.93 mm for PSO and Fa, respectively. The high *R*_*Δ*_ and *Δ* results of Table 6 mainly agree with the expected dimensions; only the *ρ* values seem a little large. However, the lower *ρ* amplitude established by PSO results in a lower *IFC*, which is in agreement with the analysis of Sect. 6.1. Moreover, although the obtained improvements are consequential, since the simulations were not repeated, and because Table 6 displays obvious differences between the dimensions, it is coherent to accept that the established dimensions do not truly describe a global optimum, but instead are efficient modifications. Finally, inserting the results of Table 6 into Eq. (16) leads to *θ*_*PSO*_ = 0.92° and *θ*_*Fa*_ = 0.73°. These angles show that while not perfectly tangent to the involute profile, the modifications tend toward a continuity order C^1^.

**Table 6:** Optimized tip relief parameters for the original gear set

	PSO		Firefly	
	Pinion	Gear	Pinion	Gear
Starting radius *R*_*Δ*_ (mm)	38.453	56.453	38.344	56.344
Curvature radius *ρ* (m)	14.033	14.033	16.902	16.902
Amount of profile modification *Δ* (μm)	45.424	45.424	37.351	37.351

**Fig. 11:**
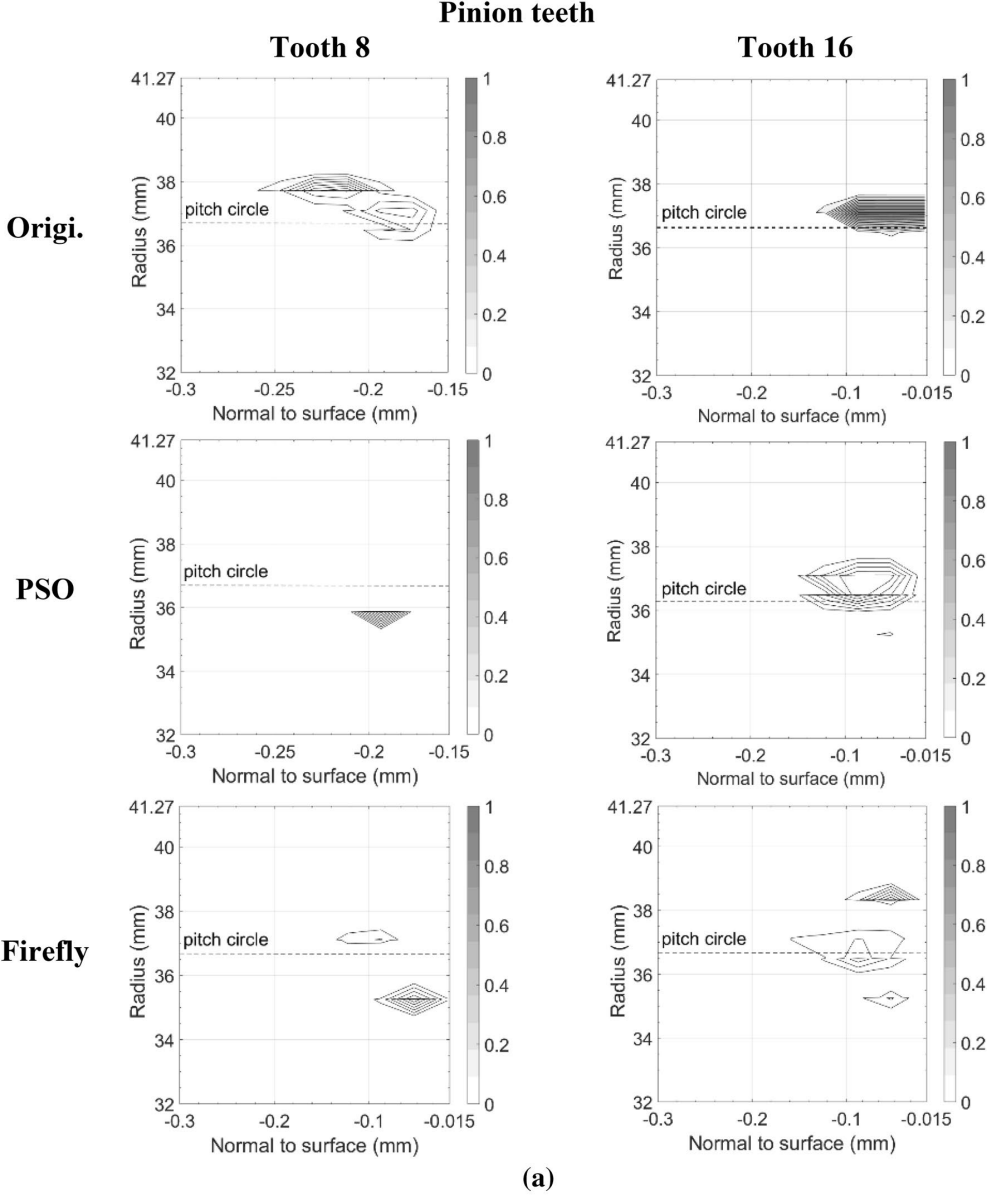

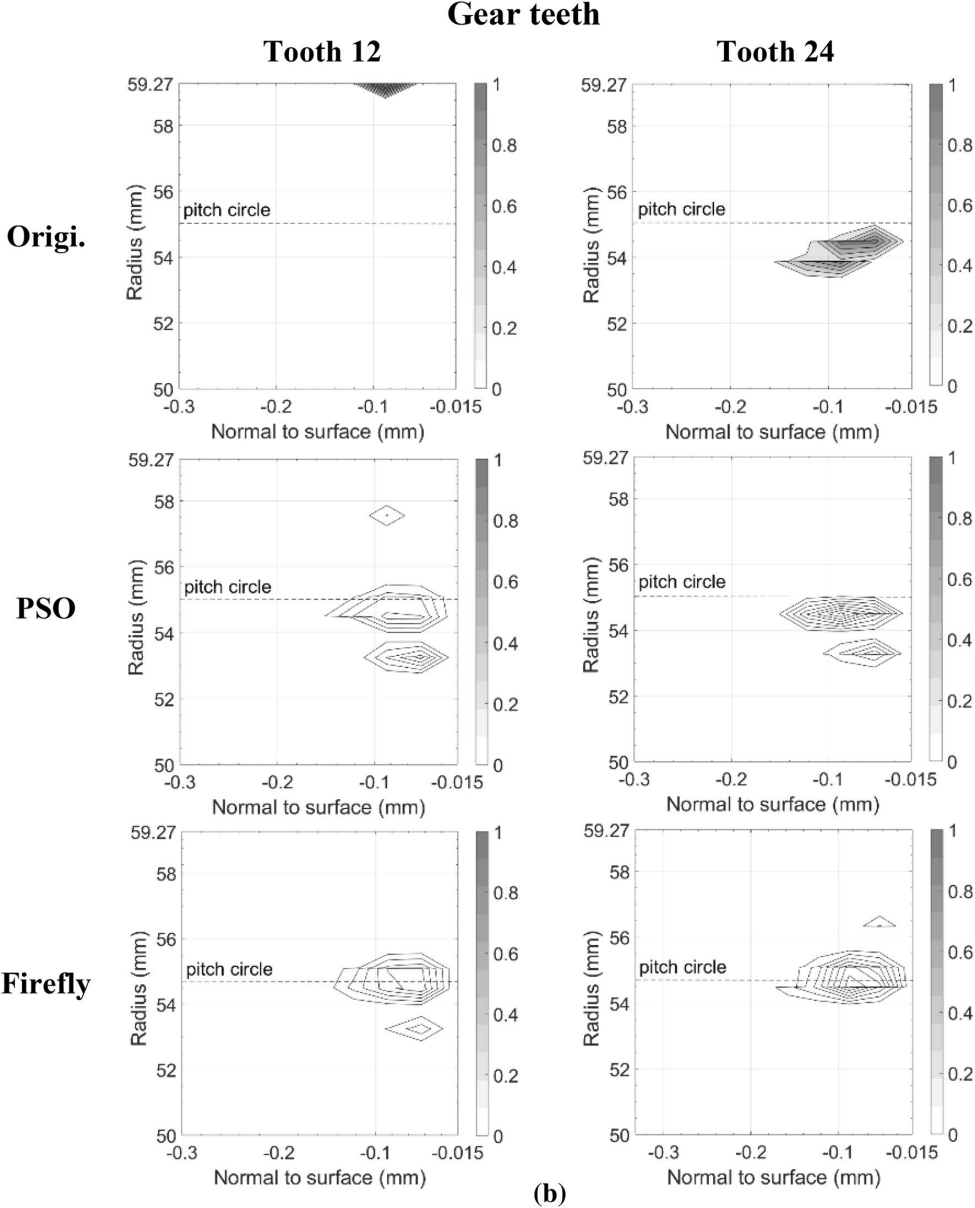
Contact fatigue initiation points after 3 × 10^5^ loading cycles at pinion speed 13,000 rpm—**a** pinion teeth, **b** gear teeth

**Fig. 12:**
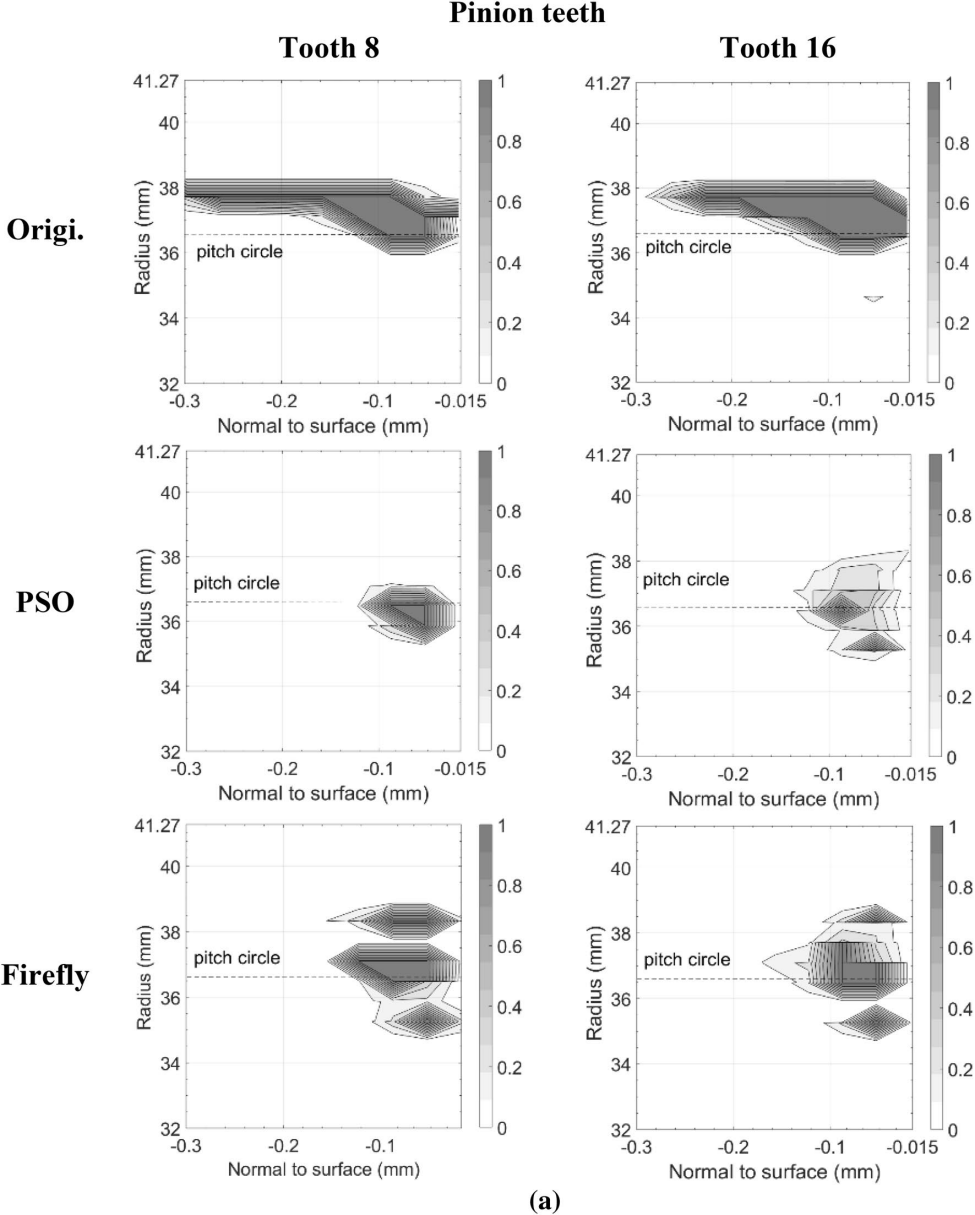

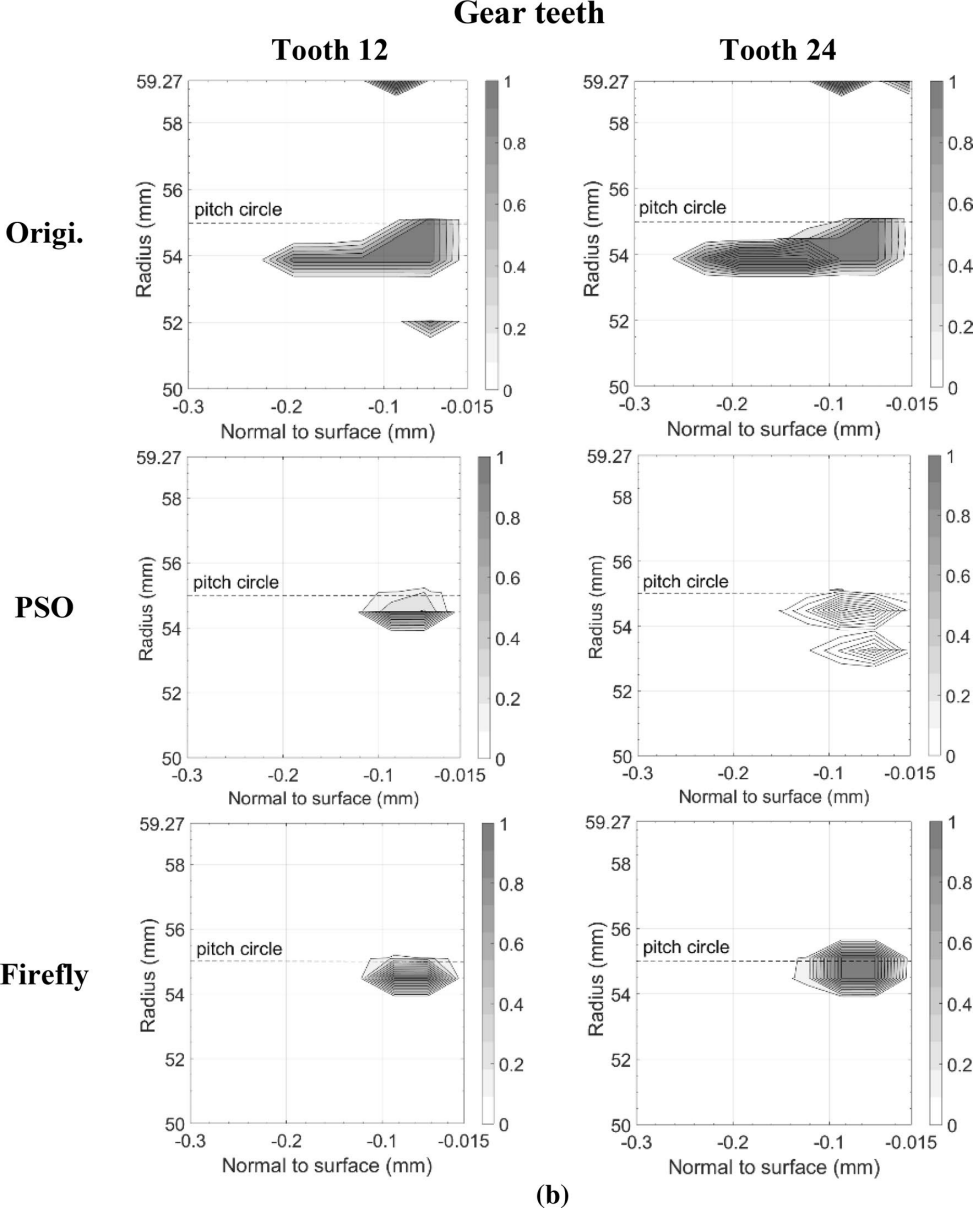
Contact fatigue initiation points after 10^6^ loading cycles at pinion speed 13,000 rpm—**a** pinion teeth, **b** gear teeth

Figures 11 and 12 present the damage accumulation *D* [defined by Eq. (4)] predicted after 3 × 10^5^ cycles and 10^6^ cycles for the pinion tooth numbers 8 and 16, and the gear tooth numbers 12 and 24, respectively. They juxtapose the responses of the original, as well as the PSO and the Fa optimum designs. These graphs depict the condition of the monitored subsurface layer described in Fig. 2. The contour lines outline the zones affected by the initiation process of contact micro-cracks. More precisely, micro-cracks are initiated solely in the positions where the damage accumulation *D* reaches a value of one.

Figure 11 indicates that after 3 × 10^5^ cycles, the accumulated damage on the original gear set is already sufficient to initiate micro-cracks beneath the surface, just above the pitch radius of pinion tooth 16 and below the pitch radius of gear tooth 24. Although the graphs cannot provide a perfect portrayal, in reality the affected zone of the gear teeth close to the tip radius suffered from plastic deformations at a depth of 87.5 μm. This area is clearly visible on tooth 12. In the graph of tooth 24 however, the tiny zone remains indiscernible. In contrast to the original gear set plots, the graphs depicting the best modifications of PSO and Fa show damage levels insufficient to initiate any contact cracks; the damage measure *D* remained below one at all surveyed points.

The graphs describing the original gear set in Fig. 12 indicate that after 10^6^ cycles, both of the considered pinion teeth contain substantial zones of initiated micro-cracks. The affected areas even reached the first row of the monitored points situated just below the surface at a depth of 17.5 μm. They also extend over the complete thickness of the investigated layer. Likewise, the gear graphs show that tooth 12 and tooth 24 now embody large regions of initiated micro-cracks, for the most part located below the pitch radius. The plots also reveal that the damaged zones remain concentrated beneath the surface at depths higher than 17.5 μm. The lower number of revolutions undergone by the gear wheel explains this condition. The areas near the tip sustaining plastic deformations also appear more clearly.

The additional loading cycles provoked the emergence of contact micro-cracks in pinion tooth numbers 8 and 16 of both the PSO and Fa optimum designs. However, compared to the original gear set, the touched regions remained smaller, did not reach the surface of the profile, and did not exceed a depth of 160.0 μm. On the other hand, while for the PSO design the damaged areas emerged concentrated around the pitch radius, for the Fa gear set version, they tend to develop a little more above the pitch radius and to cover broader regions along the profile length. In fact, the damaged spot ends at a radius equal to *R*_*Δ*_. Overall, the PSO version of the pinion seems to offer a better fatigue resistance.

The gear wheel of both PSO and Fa versions induced improvements that are even more substantial; the damaged extents concentrate slightly below the pitch radius, and did not exceed a depth of 160.0 μm, whereas the damage zones in the original gear went past a depth of 250.0 μm. Like the pinion, the gear of the PSO design exhibits impaired areas with lower damage level than the Fa version.

**Fig. 13:**
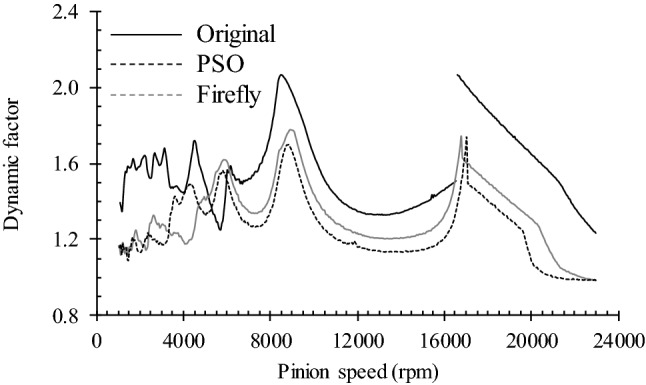
Dynamic factor of tested gear pairs with optimized profile correction

**Fig. 14:**
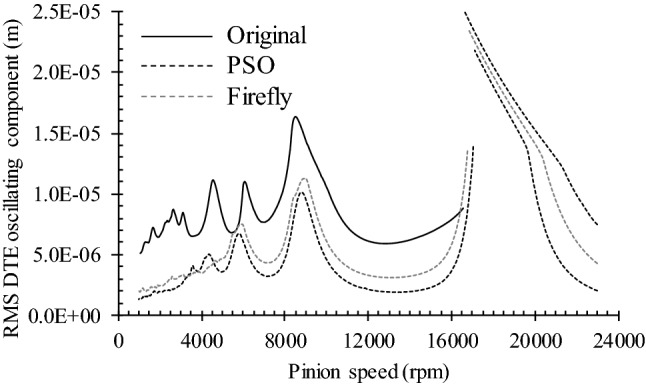
RMS of the DTE oscillating component of tested gear pairs with optimized profile correction

Since transmissions seldom operate at constant speed, the following evaluates the dynamic response of the three versions of the gear set over the speed range covered in Fig. 8 (1000 to 23,000 rpm). Figure 13 displays the dynamic factor *K*_*v*_, while Fig. 14 presents the oscillating component of the DTE. The simulations maintained the previous resistive torque of 148.4 Nm.

The chart in Fig. 13 indicates that the optimization process applied at a specific speed (here 13,000 rpm) markedly improves the gear set dynamic load over almost the complete speed range. The improvement is particularly notable in the resonance and supercritical ranges. The super-harmonic *ω*_*res*_/2 zone also reveals a significant reduction of *K*_*v*_. Only the speed range enclosing the super-harmonics *ω*_*res*_/3 and *ω*_*res*_/4 exhibits some amplification of *K*_*v*_, although the final amplitude stays below the values established at *ω*_*res*_ and *ω*_*res*_/2. This graph also sustains the previous indications suggesting that the PSO optimum version of the gear set tends to produce greater improvements than the Fa option.

The comparison of the DTE oscillating component provided in Fig. 14 leads to conclusions similar to those drawn from the results of Fig. 13. Only the resonance zone, which demonstrates DTE amplitude reductions of a lesser significance, and the super-harmonic range, which shows larger amplitude reductions, reveal different effects of the design modifications onto these distinct elements of the dynamic response.

**Fig. 15:**
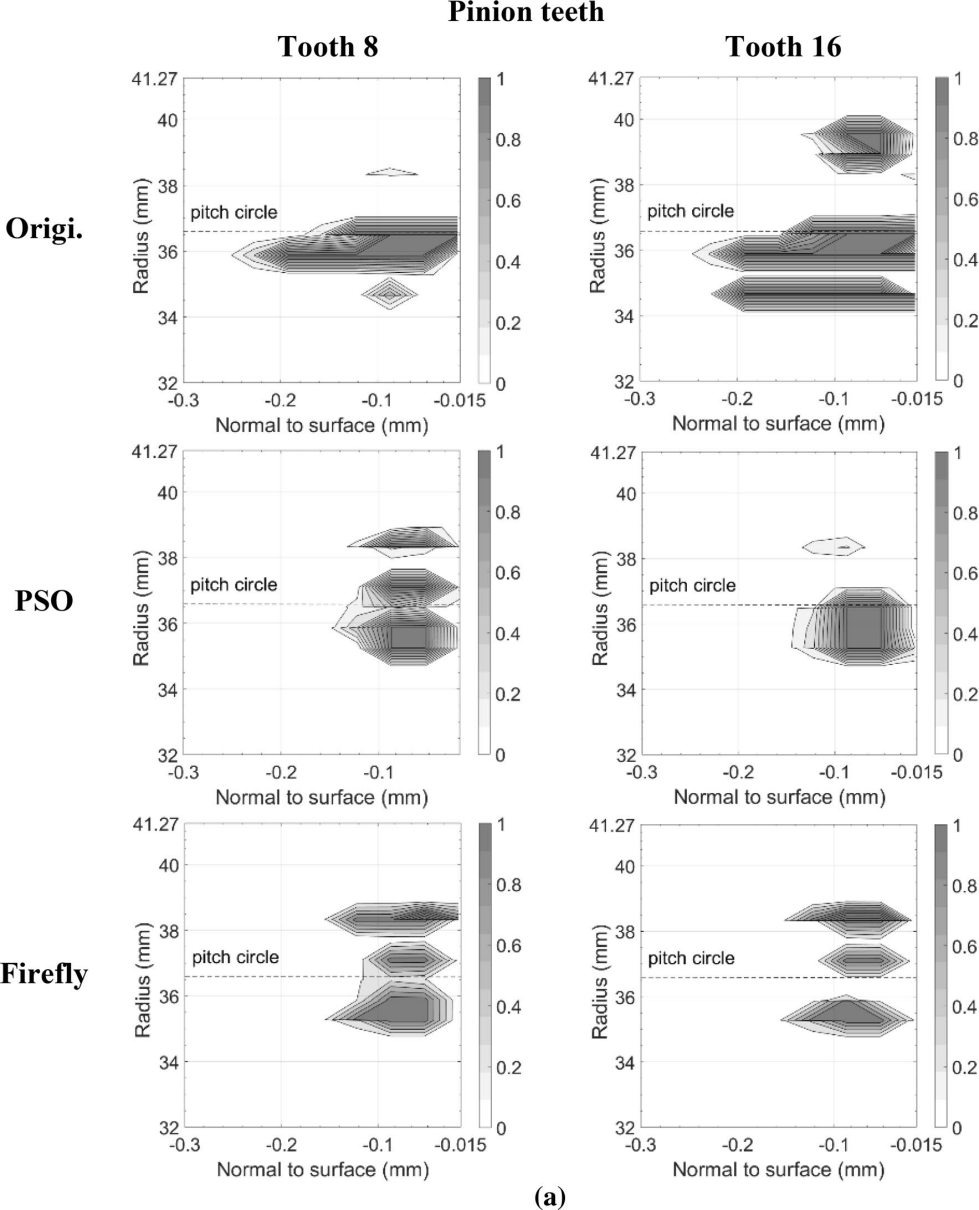

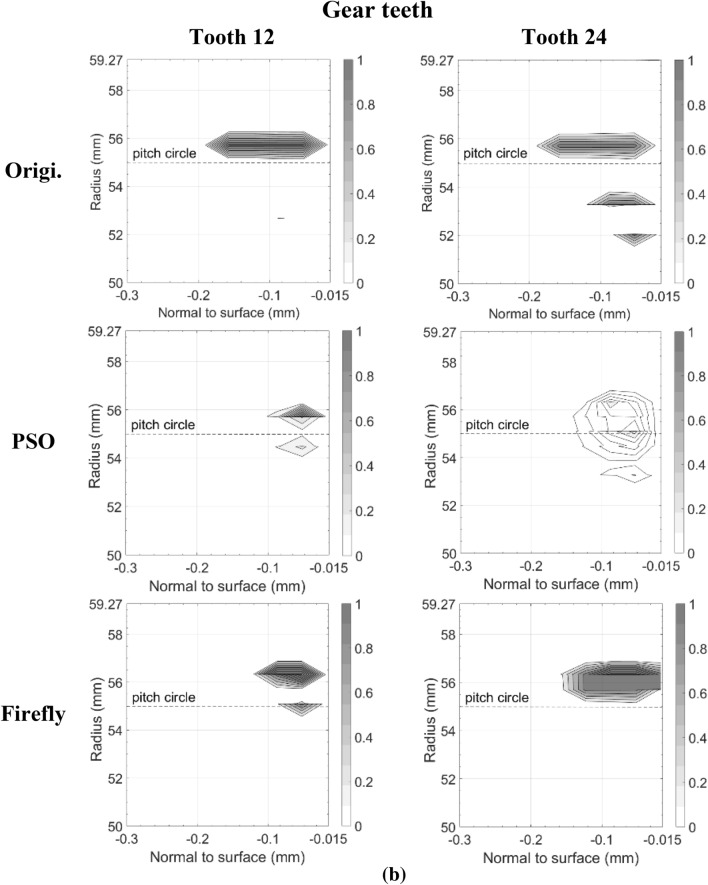
Contact fatigue initiation points after 1 × 10^6^ loading cycles at pinion speed 2500 rpm—**a** pinion teeth, **b** gear teeth

It is worth underscoring that Figs. 13 and 14 display dynamic results, but do not truly evince their aftereffects; degradation of the contact surfaces results from contact pressures and corresponding contact stresses. Therefore, to provide a more complete description of the real influence of the profile corrections, Fig. 15 displays the damage accumulated in the four teeth of Figs. 11 and 12 after 10^6^ loading cycles at a pinion speed of 2500 rpm. The graphs in Figs. 13 and 14 indicate that, compared to 13,000 rpm, this pinion speed provokes slightly higher *K*_*v*_ and DTE values for the original gear set. On the other hand, the PSO and Fa modifications lead to conditions similar to those undergone at 13,000 rpm.

The plots of the original gear set included in Fig. 15 indicate that the four teeth accumulated damages sufficient to initiate micro-cracks. As with the earlier pinion speed, the micro-cracks generated in the pinion teeth reached the first row of the monitored points at a depth of 17.5 μm. However, the damages did not extend as deep down into the teeth as under the prior conditions. Moreover, while under the 13,000 rpm pinion speed, the micro-cracks mostly developed above the pitch radius, under the current conditions they largely emerged in the dedendum portion of the teeth. The gear plots demonstrate an inverse tendency; at the 13,000 rpm inlet speed, the micro-cracks essentially arose below the pitch radius, whereas at 2500 rpm they principally developed in the addendum part. However, for the pinion teeth, the crack initiation remained close to the surface and did not exceed a depth of 262 μm. Most interestingly, the plastic deformation generated at 13,000 rpm near the tip radius almost completely disappeared. Actually, while not visible in the graph of Fig. 15, only one surveyed point situated at the tip radius of tooth 24 suffered from plastic deformations.

While the original gear set suffered lower damage levels at a pinion speed of 2500 rpm than at 13,000 rpm, the contact fatigue response of the optimized versions is more puzzling. For example, the pinion of the PSO design, which showed few micro-crack initiation points close to the pitch radius at 13,000 rpm, now demonstrates areas of initiated cracks extending along the profile length. In particular, the results reveal the occurrence of micro-cracks at the junction between the involute and the correction profiles. Conversely, compared to the previous operating conditions, the number of micro-crack initiation points markedly dropped in the gear teeth. On the other hand, the final health state of the Fa optimal version depicted in Fig. 15 declined compared to that of Fig. 12. While the 13,000 rpm speed led to the induction of micro-cracks located in the pinion teeth around the pitch radius, slightly below and above, up to the junction of the involute and correction profiles, the second tested pinion speed accelerated the crack initiation process. More unexpectedly, it even caused subsurface plastic deformations at the involute-correction profile junction. The final picture is virtually the same for the gear teeth; the crack initiation process progressed more rapidly, and subsurface plastic deformations emerged at the beginning of the correction. In sum, even though the graphs in Figs. 11 and 12 suggest that the 2500 rpm operating conditions should not be more severe than those at 13,000 rpm for both the PSO and Fa gear set versions, it appears that for the Fa design they engender considerable amplifications of the contact fatigue process. Therefore, based on these results, we have to conclude that reducing the oscillating component of the DTE generates more or less commensurate decreases of *K*_*v*_, but does not necessarily induce proportionate improvements in the contact fatigue life.

## Conclusion

This paper examined the dynamic performances of circular profile modifications, designed to optimize the fatigue life of spur gears. The first part added the PSO and Firefly metaheuristic algorithms to an existing gear dynamic/contact degradation model. In order to provide a superior management of the dynamic–fatigue computation times and efforts, this study proposed a modified version of the Firefly algorithm. This change allows the process precision to be preserved, while reducing the number of function evaluations at each iteration. A simplified evaluation of this reduction shows that it may attain up to $(n - 3)(n - 1)^{ - 1}$, which corresponds to a reduction of 92.3% per iteration when *n* is 27 particles.

The analysis of the 3^3^ factorial design formed by the initial uniform particle distribution agrees with the current literature; while the influence of the correction length is much more significant, both the profile modification amount and its length may potentially improve dynamic gear behavior. Although increasing the amount of profile correction may amplify the DTE oscillating component, when combined with moderate correction lengths, larger values reduce this dynamic measure more effectively. On the other hand, the larger amounts of profile correction associated with shorter correction lengths are more effective in reducing the number of initiation points of contact fatigue. In addition, the results obtained for the considered gear set reveals that, even though the influence of the curvature radius remains modest compared to the two other variables defining a circular modification, curved corrections are slightly more effective in ameliorating contact fatigue life, whereas larger curvature radii should better reduce the DTE oscillating component.

The last part of the paper evaluated the dynamic response of gear sets designed with corrections optimized to improve their contact fatigue life. Compared to the original gear set, the modified PSO and Firefly versions showed that optimized circular profile modifications could bring off substantial decreases in the number of initiation points of contact fatigue. Moreover, the analysis demonstrated that, even solely based on contact fatigue evaluations realized at a unique operation speed, the optimization process generates profile shapes also reducing both the DTE and the dynamic factor. The results showed as well that the profile corrections remain effective over a broad range of operation speeds. On the contrary, when operated at a lower input speed, the optimized gear sets displayed worsening fatigue degradation, despite the fact that both the DTE and the dynamic factor showed amplitudes similar to those exhibited at the optimisation speed. The pinion teeth particularly suffered from accelerated micro-crack initiation. The Firefly version of the profile even showed plastic deformations at the involute-correction profile junction in both the pinion and the gear teeth. On the other hand, at this lower speed, the original gear set displayed reduced damage levels, whereas the corresponding DTE and dynamic factor demonstrated slight increases.

## Data Availability

All input data considered during the simulations and necessary to reproduce the results are included in Tables 1, 2 and 3. Therefore, no additional data is appended.

## Code availability

The results presented in Figs. 8, 9, 10, 11, 12, 13, 14 and 15 were obtained from a combination of the optimization algorithms detailed in Figs. 4, 5 and 6 and the gear contact model published in [18]. The flowchart of Fig. 1 also describes this gear model. Therefore, no additional code is appended.
